# Comparison of performance of medical students between two teaching modalities “Flip the classroom” and traditional lectures: A single center educational interventional study

**DOI:** 10.12669/pjms.36.5.2730

**Published:** 2020

**Authors:** Fadwa Tahir, Bayan Hafiz, Taghreed Alnajjar, Bayan Almehmadi, Bayan Besharah, Abdulrahim Gari, Yasir Katib

**Affiliations:** 1 Dr. Fadwa Tahir, FRCS Canada., Assistant Professor, Department of Obstetrics and Gynecology, Faculty of Medicine, Umm Al-Qura University, Makkah, Saudi Arabia; 2 Dr. Bayan Hafiz, MBBS. Resident, Department of Laboratory Medicine and Pathology, King Abdul-Aziz Medical City, Jeddah, Saudi Arabia; 3 Dr. Taghreed Alnajjar, MBBS. Resident, Department of Ophthalmology, Security Forces Hospital, Riyadh, Saudi Arabia; 4 Dr. Bayan Almehmadi, MBBS. Resident, Department of Pediatric Medicine, Prince Sultan Military Medical City, Riyadh, Saudi Arabia; 5 Dr. Bayan Besharah, MBBS. Resident, Department of Otorhinolaryngology Head & Neck Surgery King Fahad Armed Force Hospital, Jeddah, Saudi Arabia; 6 Dr. Abdulrahim Gari, FRCS Canada. Consultant, Department of Obstetrics and Gynecology, King Faisal Special Hospital and Research Center, Jeddah, Saudi Arabia. Assistant Professor, Department of Obstetrics and Gynecology, Faculty of Medicine, Umm Al-Qura University, Makkah, Saudi Arabia; 7 Dr. Yasir Katib, FRCS Canada. Consultant, Department of Obstetrics and Gynecology, Sulaiman Faqih hospital, Jeddah, Saudi Arabia. Assistant Professor, Department of Obstetrics and Gynecology, Faculty of Medicine, Umm Al-Qura University, Makkah, Saudi Arabia

**Keywords:** Gynecology, Medical student, Obstetrics

## Abstract

**Objectives:**

This study aims to compare the students’ performance in Obstetrics and Gynecology by using two teaching modalities, i.e., Flip the classroom (FTC) compared to Traditional lectures (TL) among final year medical students and assessment of the students’ satisfaction towards FTC as learning modality.

**Methods:**

An educational interventional study was conducted on 136 females final year medical students at Umm Al-Qura University, Makkah, Saudi Arabia from September to December; 2017. Out of 40 core topics of Obstetrics and Gynecology, eight were chosen for FTC and eight for TL. The performance in each teaching modality was assessed by comparing the score of the students in multiple choice question (MCQ) and objective structured clinical examination (OSCE) in the final examination. The final performance was compared between the FTC and TL selected topics. The data was analyzed by using SPSS version 16 (SPSS Inc., Chicago, IL, USA).

**Results:**

MCQ and OSCE grades of students (n=136) were significantly higher in FTC versus TL topics, i.e., mean ± standard deviation (13.4 ± 2.7 vs. 12.3 ± 2.4; p < 0.001) and (33.9 ± 4.3 vs. 30.4 ± 4.7; p < 0.001), respectively. Almost 60% of the students expressed their satisfaction with FTC modality.

**Conclusion:**

Scores were significantly high for Flip the classroom topics compared to Traditional lectures.

## INTRODUCTION

University level education has been executed in a comparatively analogous method since long time and across the cultures. The traditional lectures (TL) by a professor (a central pillar), transferred information to recipient students, is one directional way of transmitting the knowledge.^1^ Though, university education with TL have been criticized over the past 30 years. The main points of criticism are the following: students’ do not have the mechanisms on firming academic engagement with the study material because they became more inert in TL; student’s responsiveness declines speedily; the pace of the lectures is not adjusted to all the students needs and TL not suited for teaching higher directive skills such as application and analysis.^2^-^4^

Therefore, various scientists and educationalists have endorsed different forms of lecturing based on active learning philosophy, i.e., involvement of innovative technology mediated collaborations and enhanced lectures.^2^,^5^,^6^ However, despite the comprehensive critique, the TL continues to prevail as the chief moralistic strategy in higher education.^7^

Teachers of undergraduate studies are trying their best to plan courses that can help to develop deep and active learning approaches in their students. According to educational psychologists, undergraduate students should be helped to avoid surface learning tactics especially the mere memorization of subject content in order to ascertain good scores on examinations. Students should be encouraged to in-depth learning tactics that are described by initiative to understand basic ideologies and perceptions by dealing expressively with content. A new educational method that is professed to support this educational phenomenon in basic medical science disciplines is the flip the classroom (FTC).^8^

Therefore, an immense need of medical education to be transformed in required.^9^,^10^ Competent physicians are one of the most important healthcare resources in order to deliver the healthcare with safety, efficiency and effectiveness. These demands of healthcare require medical education to be reformed along with the implementation of learner-centered prototypes and competency-based syllabuses in which student development is realized by demonstration of academic content irrespective of time, place, and pace of learning.^9^-^11^

Social networks, video recording and mobile devices are allowing educators at all levels to flip their classrooms to meet the needs of this time.^12^ Moreover, the medical educators will have prospects to change longitudinal and inter-professional education experiences if they are sensitive to the organizational change required for this new modality of learning.^13^

In FTC model, “students are required to participate in pre-class preparatory work, which may include the use of prerecorded lectures, readings, or online modules. Class time is then repurposed to focus on problem solving, application, synthesis, and collaborative learning”.^8^

This study aimed to compare the performance among 5th year female medical student at Umm Al-Qura University (UQU) between two teaching modalities, i.e., FTC and TL in Obstetrics and Gynecology (Ob/Gynae) topics.

## METHODS

An educational interventional study by using split plot design was conducted on 136 females medical 5^th^ year students at UQU, Makkah, Saudi Arabia. The study period was from 10^th^ of September to 7^th^ of December; 2017. Out of 40 core topics of Ob/Gynae, eight were chosen for FTC and eight for TL evaluation.

The Ob/Gynae department divided the topics into two groups based on final weight (FW) by using the following equation:

Final Weight of each topic *(FW) = W ÷ ΣW*

Σ = sum of all

*W = I X F*.

*I* = impact of the underlined problem in the population present within the topic.

*F* = frequency of occurrences of underlying problemin the population present within the topic.

The *FW* has been calculated to create equal weight of two teaching modalities in the context of importance of topics and their value in the final exam question distribution. This helps in increasing the internal validity of the results.

Impact and frequency of each topic has been searched by literature review. Impact has three categories and each category has been given a weightage, i.e., non-urgent, little prevention potential = 1; serious, but not immediately life threatening = 2; and life -threatening emergency and/or high potential for prevention impact = 3. Similarly, frequency has been categorized into three levels based upon the weightage, i.e., rarely seen = 1; relatively common = 2; and very common = 3.^13^

**Fig.1:**
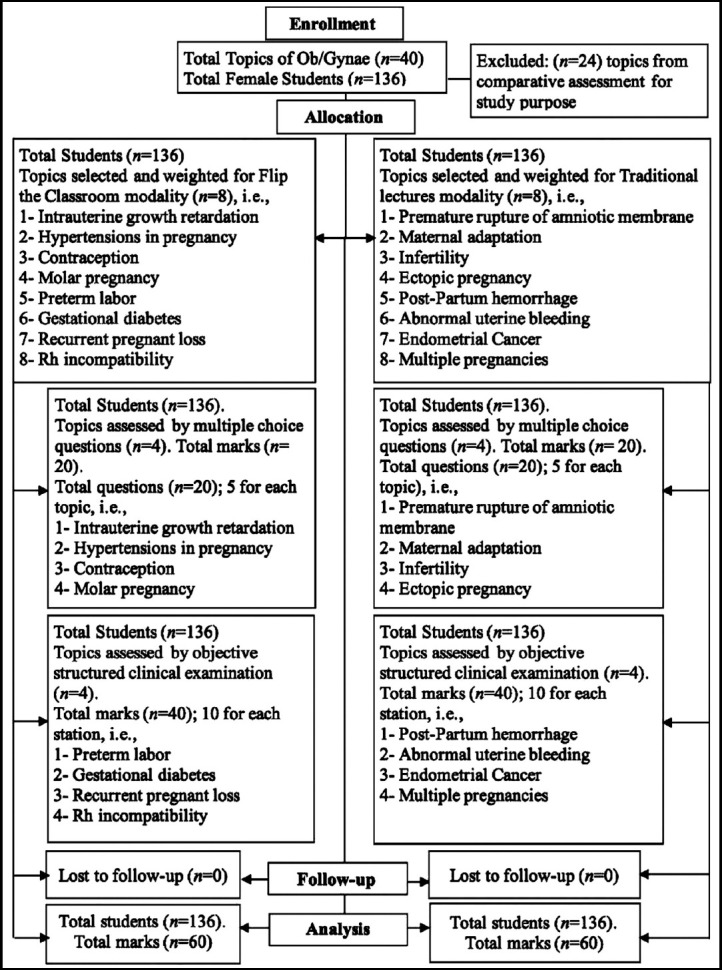
Consolidated standards of reporting trial (CONSORT) flow diagram.

Each modality has eight topics which are equal in FW. The selected eight topics in FTC modality group were; intrauterine growth retardation (FW=3), hypertensions in pregnancy (FW=3), contraception (not included for FW calculation), molar pregnancy (FW=2), preterm labor (FW=5), gestational diabetes (FW=5), recurrent pregnant loss (FW=3) and Rh incompatibility (FW=3) while in TL group topics were premature rupture of amniotic membrane (FW=5), maternal adaptation (not included for FW calculation), infertility (FW=2), ectopic pregnancy(FW=3), post-partum hemorrhage(FW=5), abnormal uterine bleeding (FW=3), endometrial cancer (FW=3) and multiple pregnancies (FW=3) (Fig.1).

The students were taught for a total of 13 weeks followed by final examination and assessment. After the final examination, each student was sent a statement to determine the satisfaction level with FTC as a teaching modality via personal email messages with the option of five-point Likert scale, i.e., “I am satisfied with Flip the classroom as a new teaching modality”. Likert scale was started from strongly disagree, i.e., “1” to strongly agree “5”.

For FTC topics, students received the lectures in the form of videos and recorded electronic power point material attached to reading materials. After the student read and watched the material, an assignment was given to him. All study material was given to the students a week before the day of discussion to have enough time preparing and solving the assignments on the scheduled day. On the day of discussion, the students are divided into small groups of 18 students with a mentor in each. The role of the mentor is to guide the discussion and answering for the unsolved queries raised by students.

In TL were held in the classroom where all students attend and listen the lecture with power point presentation followed by the usual questions answers session. Student performance during the Ob/Gynae course evaluated by multiple choice questions (MCQ) and objective structured clinical examination (OSCE) that assessed the knowledge and clinical skills, respectively. MCQs assessment was done for eight topics (four for FTC and four for TL) by 20 MCQ questions in final assessment (5 questions for each topic). Rest of the eight topics were assessed by OSCE. There were 10 OSCE stations, i.e., four for FTC and four for TL topics each (maximum marks for each station was 10). There were two rest stations among the 10 OSCE stations. The marks obtained by the students from FTC topics were compare to TL in both OSCE, MCQs and overall (OSCE + MCQs). Candidates who attained cumulative overall marks < 60% for FTC and TL topics each are considered as failed (Fig.1).

The data was analyzed by using SPSS version 16 (SPSS Inc., Chicago, IL, USA). Students satisfaction was measured by number and percentage. Normality of variables, i.e., mean difference (MD) of the scores of MCQs, OSCE, and overall (MCQs + OSCE) between FTC vs. TL was confirmed by Shapiro-Wilk test, skewness and kurtosis. The *p* value > 0.05 was considered supportive for normality in Shapiro-Wilk test. Maximum and minimum allowable values of both skewness and kurtosis was considered as -1 to +1 for *t*-test.^14^ Normally distributed data was expressed in mean ± standard deviation (mean ± SD). MCQs, OSCE and overall scores for FTC and TL were compared by using paired *t*-test followed by calculation of 95% confidence interval (CI) of mean differences (MD). The effect size of each paired *t*-test was also estimated by calculating Cohen’s *d*. The effect size has been categorized low, medium and high if Cohen’s *d* was found to be 0.2-0.49, 0.5-0.79 and >=0.8, respectively).^15^ *Post-hoc* power analysis was done by using Stata version 14.^16^

Informed consent was ascertained from the students after they had been made aware of the study rationale and objectives along with reassurance of keeping the confidentiality of their results. Ethical approval for this study has been sought from the Bio-ethical committee of UQU (Approval No. HAPO-02-K-012-09-58, dated Sept. 8, 2016), Makkah, Saudi Arabia. Authors have not declared any conflict of interest. No source of funding was available and declared.

## RESULTS

One hundred and thirty-six female students were involved in the study. The MD of MCQs, OSCE and overall was normally distributed because all have *p* value of Shapiro-Wilk test > 0.05. The MD of the MCQs scores for FTC vs. TL showed skewness and kurtosis at -0.005 and 0.25, respectively. The mean score for MCQs for FTC and TL topics were 13.4 ± 2.7 and 12.3 ± 2.4, respectively with the MD of 1.07 with 95% CI of 0.63 to 1.51 and p value < 0.0001. However, the Cohen’s d was 0.42. The MD of the OSCE scores for FTC vs. TL showed skewness and kurtosis at 0.003 and -0.55, respectively. Mean score for OSCE for FTC and TL topics were 33.9 ± 4.3 and 30.4 ± 4.7, respectively, with MD of 3.6 with 95% CI of 2.9 to 4.3 and p < 0.0001. However, the Cohen’s d value was 0.9. Overall (MCQs + OSCE) scores for FTC vs. TL (MD) showed skewness and kurtosis at -0.36 and 0.32, respectively. The mean score for OSCE for FTC topics was 47.3 ± 6.1 and for TL topics was 42.8 ± 5.9. The MD was 4.7 with 95% CI of 3.8 to 5.5; i.e., p < 0.0001. Cohen’s *d* was 0.95 (Table-I).

**Table-I:** Comparative analysis of final evaluation between Flip the Classroom and Traditional Lectures teaching modalities.

Mode of Assessment	n	FTC	TL	Mean Difference	t statistics	df	p-value
MCQs (FTC vs. TL)	136	13.4 ± 2.7	12.3 ± 2.4	1.07 (0.63 - 1.51)	4.8	135	0.000004
OSCE (FTC vs. TL)	136	33.9±4.3	30.4 ± 4.7	3.6 (2.9 - 4.3)	10.5	135	<0.0001
Overall (FTC vs. TL)	136	47.3±6.1	42.7 ± 5.9	4.7 (3.8 - 5.5)	11.02	135	<0.0001

**n:** Number of students; **FTC:** Flip the classroom; **TL:** Traditional lectures; **df:** Degree of freedom;

**MCQs:** Multiple choice questions; **OSCE:** Objective structured clinical examination.

About 58.5% of students scored overall of ≥80% in FTC topics comparing to 26.5% of TL topics while 14% of students scored less than 60% in TL topics comparing to 5.2% of FTC topics.

Out of 136 students, 108(79%) answered the satisfaction statement, and 60% of them checked the points, i.e., agree and strongly agree. Power of 99% was estimated by keeping the study subjects of 136, null hypothesis of zero mean difference, 1.08 as of actual mean difference of MCQs scores between FTC vs. TL topics with standard deviation of 2.6. Type one error probability associated with this null hypothesis was considered as 0.05. The mean difference of OSCE and total scores between FTC vs. TL topics were higher than that of MCQs, so showed higher power than that of calculated for MCQs mean difference.

## DISCUSSION

According to our results, students scored high in FTC topics as compared to TL topics in both methods of assessment, i.e., MCQs, OSCE and combined MCQs with OSCE. Similar result was found in a quasi-experimental study which was done among the nursing students to compare between three learning approaches: traditional lecture only, lecture and lecture capture back-up, and the FTC approach to lecture capture with innovative classroom activities. The examination scores results were higher for the FTC group (81.9) compared to the other groups where lecture and lecture capture back-up group (80.7) and the lowest was in lecture only group (79.8).^17^ Not only in healthcare sector, in a study of computer programming course where students who have been taught by FTC modality attained higher score as compared to TL. Furthermore, the better performance was sustained for 3 years (full duration of study).^18^ A study conducted on pharmacy students where the FTC teaching modality included active-learning activities and formative assessments which transmuted the classroom interfaces of not only the teachers but also the students that lead to improved students’ examination performance.^19^ A meta-analysis of comparative studies (between-subject design) showed an overall significant effect in favor of FTC vs. TL for health professions education (standardized mean difference, SMD = 0.33, 95% confidence interval, CI = 0.21-0.46, p < 0.001). Furthermore, it was found that FTC approach became more effective by instructors who used questions at the beginning of each in-class session.^20^

In our study we found lower effect size of MD for MCQs but for OSCE and overall (MCQs and OSCE) it was large, i.e., > 9 that showed the assessment by OSCE and overall in FTC topics scored > 0.90 standard deviations higher on assessment than that of TL topics. This difference in effect size may be due to the factor that students might have performed better in FTC of OSCE as compared to MCQs. On the other hand, this technique is more effective in clinical skills improvement assessed by OSCE rather than knowledge which has been assessed by MCQs. Effect size quantitatively measures the magnitude of the interventional effect. It determined the strength of the relationship between two variables, i.e., larger effect size means stronger relationship.^15^

Our study results regarding students’ satisfaction are nearly consistent with other studies, i.e., 75% of students appreciated FTC in the study of Nouri J and cumulative of 71% students were satisfied with FTC in five different studies analyzed in a meta-analysis.^20^,^21^ However, combination of new teaching skills with interactive classroom activities can improve the learning but not inevitably improve student satisfaction as emphasized in one study where students did not perceive the value of interactive learning tactics and enforced the need of more effort on this approach applicability.^17^ Benner et al. illustrated that student satisfaction may not be a reliable measure of learning.^22^

FTC can be a solid base of curricular transformation in medical education. It can be implemented on a single lecture or to the complete curriculum. However, considerable modifications in the management of stratagems including administration of thorough medical education research by new teaching approaches and competency-based didactic outcomes are required to fully realize the potential of the FTC.^23^

### Strengths of the study

One strength of this study is that comparison of the study modalities is within the same person that removed the effect of between-person variability and hence have a more powerful analysis. In other words, we are using person as a matching factor, which means that each person acts as their own control. Another strength is the post-hoc power calculation of the study that was found > 95% reflecting good level of precision in estimates. This showed that the sample was enough to reject the false null hypothesis or less risk for Type-2 error and more chances to find a difference when assuming real difference. Results were precise because of narrower 95% CI for mean differences in MCQs (0.63-1.51) and OSCE (2.9-4.3). No missing data was found that also improved the study power.

### Limitations of the study

This work revealed that FTC modality improved the students understanding of the topics assessed by final scores but there are limitations. This study has a question mark on its generalizability, as it is a single center study and selected only one gender with only one subject, i.e., Ob/Gynae. The results could be much generalizable if the study design would be cluster randomized (restricted stratified) educational interventional control trial with parallel design with multiple subjects and both genders. Another limitation is that no evaluation or monitoring of the students’ actual total time engaged with the pre-class material among FTC topics. More the time students engage with pre-class material may have higher score in evaluation for FTC. Moreover, the students who struggled to get good scores in other than FTC topics may also get good scores in the FTC topics evaluation. Students attendance for TL topics was not monitored as it might have an association with final scores. These factors could create a probability of residual confounding effect. Moreover, study objectives were explained and intervention blinding for examiners, mentors, students and data analysts could not possible due to the nature of the study. This lack of blinding could cause information bias. The implementation and in-class activities of the FTC vary greatly as described in different studies. Therefore, the methodology of FTC adopted by the student in this study may not be generalizable to other FTC designs that do not exactly follow the same procedure. Mentors knowledge, behavior and responsiveness to the students queries in FTC group should also be considered at the time of study follow-up because it may affect the final scores of the students.

## CONCLUSIONS

Current study suggests that flip the classroom methodology in medical education overall produces a statistically significant improvement in learner performance in final examination compared with traditional teaching approach. Moreover, a substantial drop in failure was observed among the students for the topics taught by flip the classroom as compared to traditional lectures.

### Recommendations

A multicenter research about FTC modality perception, usability and acceptance by both educators and students is required. Future research can be conducted to examine the possible effect of specific types of teaching method or presentation on student learning that should also examine the possible impact of video styles. Nevertheless, the increasing fame of using video-recorded lectures, we still don’t know much about influence of varied video styles on student learning. It is also required to examine whether the FTC teaching modality can promote knowledge retention over a long time period.

### Authors’ Contribution

**FT** protocol development, decide on subject’s eligibility, final approval of the version to be published

**BH** data collection, record review, protocol development, introduction section writing/review

**TA** protocol development, record review, performed informed consent process, methodology section writing/review.

**BA** data analysis, results interpretation, results section writing/review.

**BB** drafting the article, results section review and interpretations, discussion section review.

**AG** protocol development, conception and design, revising it critically

**YK** substantial contribution to conception and design, revised it critically

**FT** is responsible and accountable for the accuracy or integrity of the work.
